# Functional relationship between photosynthetic leaf gas exchange in response to silicon application and water stress mitigation in sugarcane

**DOI:** 10.1186/s40659-021-00338-2

**Published:** 2021-05-01

**Authors:** Krishan K. Verma, Xiu-Peng Song, Chhedi Lal Verma, Zhong-Liang Chen, Vishnu D. Rajput, Kai-Chao Wu, Fen Liao, Gan-Lin Chen, Yang-Rui Li

**Affiliations:** 1 Key Laboratory of Sugarcane Biotechnology and Genetic Improvement (Guangxi), Ministry of Agriculture and Rural Affairs/ Guangxi Key Laboratory of Sugarcane Genetic Improvement/ Sugarcane Research Institute, Guangxi Academy of Agricultural Sciences/ Sugarcane Research Center, Chinese Academy of Agricultural Sciences, Nanning,, 530007 Guangxi China; 2 Irrigation and Drainage Engineering, ICAR-Central Soil Salinity Research Institute, Regional Research Station, Lucknow, 226005 India; 3 College of Agriculture, Guangxi University, Nanning, 530004 Guangxi China; 4 Academy of Biology and Biotechnology, Southern Federal University, Rostov-on-Don, 344090 Russia; 5 Institute of Biotechnology, Guangxi Academy of Agricultural Sciences, Nanning, 530 007 Guangxi China

**Keywords:** Photosynthetic leaf gas exchange, Bio-modelling, Silicon, Sugarcane, Water stress

## Abstract

**Background:**

Water stress is one of the serious abiotic stresses that negatively influences the growth, development and production of sugarcane in arid and semi-arid regions. However, silicon (Si) has been applied as an alleviation strategy subjected to environmental stresses.

**Methods:**

In this experiment, Si was applied as soil irrigation in sugarcane plants to understand the mitigation effect of Si against harmful impact of water stress on photosynthetic leaf gas exchange.

**Results:**

In the present study we primarily revealed the consequences of low soil moisture content, which affect overall plant performance of sugarcane significantly. Silicon application reduced the adverse effects of water stress by improving the net photosynthetic assimilation rate (*A*_net_) 1.35–18.75%, stomatal conductance to water vapour (gs) 3.26–21.57% and rate of transpiration (E) 1.16–17.83%. The mathematical models developed from the proposed hypothesis explained the functional relationships between photosynthetic responses of Si application and water stress mitigation.

**Conclusions:**

Silicon application showed high ameliorative effects on photosynthetic responses of sugarcane to water stress and could be used for mitigating environmental stresses in other crops, too, in future.

## Background

Sugarcane (*Saccharum* spp. Hybrid) is a major C_4_ cash crop predominantly grown in arid and semi-arid areas and is an important agro-industrial crop used for various purposes, i.e. sugar and ethanol production. Nowadays, water deficit causes severe loss to sugarcane production to various countries located in arid and semi-arid zones [1, 2]. The maximum growth temperature for the highest cane productivity is about 25–30 ºC and low temperature (15–12 ºC) reduces the plant development, cane and biomass production [3, 4]. China is the third largest sugar producer after Brazil and India among the first 10 sugarcane producing countries in the globe [5]. Brazil is the largest sugarcane producer globally, and its production is mainly for ethanol and sugar productivity [6, 7]. The use of ethanol from renewable sources is mainly useful to minimize greenhouse gas emissions and dependence on fossil fuels [8].

Water stress is one of the serious abiotic stresses, which inhibits plant growth, development and production in all over the globe [9, 10]. It brings a broad range of morphological, physiological and metabolic disruptions in plants. It also accelerates the rate of leaf necrosis, chlorosis, senescence and reduce photosynthetic pigments, which in turn downregulates photosynthetic activities, leaf area-expansion, resulting in lower biomass and productivity and sometimes in total mortality of the crops [9, 11, 12]. Plants have evolved various mechanisms to counteract the negative effects of water-deficit and protect themselves against the detrimental effects of ROS over production. The most significant antioxidative defence mechanisms, comprised to enzymatic and non-enzymatic activities that effectively scavenge the ROS and maintain the proper balance within the cell activities [1, 13, 14].

Nevertheless, severe stress for a long time would bring serious losses and cells mortality may occur [14, 15]. Such circumstances necessitate advance alleviation technologies to protect water stress damage to the sugarcane productivity in the near future. Mitigation of various environmental stresses, newly developed technologies need to be discovered and applied. Nowadays, the main focus of agricultural practices is to enhance crop production, environmental stresses which occur unpredictably and cause the major loss to crop yield are not often considered. To solve this problem, newly low cost effective techniques will be useful for agro-farming systems.

Silicon (Si) has been found to have a significant potential application in agricultural crops [10, 16–18]. In various crops, during environmental stresses, application of Si plays a significant role in plants are well recognized [10, 16, 18–22]. Silicon is the eighth most abundant element in the world and second most abundant in the earth crust following oxygen on the basis of mass (28.8%) [17, 23]. Demonstration into the role of Si on plant tolerance to unfavourable environmental conditions [24] has updated our knowledge of the possible impacts of Si on plants at the molecular, physiological and ecological levels [25–27]. Reduction of oxidative damage via reduced ROS production and/ or enhanced level of antioxidative enzyme activities to play significant role in Si-induced stress mitigation [18, 28–30].

Hence, the main objective of this study was to understand the mitigation strategy of different Si application for water stress tolerance in sugarcane, with a main focus on photosynthetic leaf gas exchange. Furthermore, we aimed to identify the appropriate concentration of Si that can improve and/ or maintain the photosynthetic capacity of sugarcane during water stress.

## Materials and Methods

### Plant material and growth conditions

The experiment was carried out in an open greenhouse of Sugarcane Research Institute, Guangxi Academy of Agricultural Sciences, Nanning, Guangxi, China (22.49ºN, 108.18ºE). Newly released drought tolerant sugarcane (*Saccharum officinarum* L.) cultivar Guitang 42 (GT 42) was selected. One bud cane setts were planted in the field according to the farmer’s standard practices. After 30-days of sowing, germinated healthy plants were shifted in plastic pots (width 33 cm and height 30 cm) filled with fertile soil. The physio-chemical parameters of the applied soil are presented in Table 1. Cultivar GT 42 was developed and provided by Sugarcane Research Institute, Guangxi Academy of Agricultural Sciences, Nanning, Guangxi, China. After transplanting, the seedlings were allowed to proper establish for sixty-days. During this period, seedlings were fertilized and disease management was carried out according to commercial cultivation practices. Throughout the normal growth period, plants were watered till the filled capacity to maintain the equal volume of soil moisture. To maintain adequate soil nutrient level, i.e. nitrogen (N), potassium (P) and phosphorous (K) were applied to the soil at specific time period. The basal dose of fertilizer in soil as nitrogen (N, 70 mg kg^−1^ soil), potassium (P, 50 mg kg^−1^ soil) and phosphorous (K, 100 mg kg^−1^ soil) were applied in each pot. During the experiment, randomly uniform plants were selected for water stress (moderate, 55–50% of soil moisture) and silicon application (0, 100, 200 and 500 mg L^−1^), applied as soil irrigation. The Si solution was freshly prepared in running tap water. The Si solution was applied once in 1 month upto 4 months (treatment frequency @ 4). Calcium metasilicate (CaO.SiO_2_) was used as a source of Si. The water-stressed plants were selected as control (without Si) in the whole experiment. The soil moisture (%) was observed by Soil Moisture Meter at regular basis. The atmospheric variables such as ambient air temperature (ºC), air relative humidity (%) and sunshine (h) were recorded throughout the study (Table 2).

**Table 1:** Physio-chemical characteristics of applied soil

Parameters	Soil texture	Soil pH	Organic carbon (%)	Total N (%)	Available P (mg kg^−1^)	Available S (mg kg^−1^)	Exchangeable K (cmol( +)kg^−1^)	Available Si (mg kg^−1^)	Available Cu (mg kg^−1^)	Available Fe (mg kg^−1^)
Value	Silty-clay	5.9	0.72	0.09	9.18	12.01	2.71	87.3	0.85	12.0

**Table 2:** Atmospheric variable during experiment

		March	April	May	June	July	August	September
Ambient air temperature (ºC)	Maximum	25.5	27.6	30.4	32.1	32.9	32.7	31.6
Minimum		15.0	19.6	22.8	24.9	25.4	25.2	23.6
Mean		20.3	23.6	26.6	28.5	29.2	29.0	27.6
Ambient air humidity (%)	Mean	82	81	80	82	82	78	75
Sunshine (h)	Mean	12	12.7	13.2	13.5	13.4	12.9	12.3

### Leaf gas exchange measurements

The leaf gas exchange was measured on the 30, 60, 90 and 120 days after water stress with and without Si application (0, 100, 200 and 500 mg L^−1^). Net photosynthetic assimilation rate (*A*_net_), stomatal conductance to water vapor (gs) and rate of transpiration (E) were observed by portable photosynthesis system (LI-6800, LI-COR Biosciences, Lincoln, NE, USA) from photosynthetically fully mature leaves (leaf + 1). From each treatment, a minimum of five (*n* = 5) measurements were recorded between 9:00 and 11:30 h. Photosynthetic active radiation (PAR), temperature and CO_2_ concentration was adjusted to 1200 µmol m^−2^ s^−1^, 25 ºC and 400 ppm in the leaf chamber while observing photosynthetic capacity.

## Bio-modelling of net photosynthetic assimilation rate, stomatal conductance and transpiration rate in response to silicon application for water stress mitigation

### **Hypothesis-I**

Rate of change of physiological response (*A*_net_, gs and E) with respect to silicon application (dPs_i_/dSi) is directly proportional to the appropriated physiological response rate against silicon dose. Mathematically it can be expressed as below.

1 $$\frac{dPSi}{{dSi}}\,{\kern 1pt} {\kern 1pt} \propto {\kern 1pt} {\kern 1pt} \,\frac{P - \alpha }{\beta }$$

*P*_Si_ = physiological response rate against applied doze of silicon.

α, β = appropriation constants.

Instead of minus sign in Eq. 1 positive sign could be also used.

Equation 1 Can be also written as below after introducing a proportionality constant (γ)

2 $$\frac{dPSi}{{dSi}}\,{\kern 1pt} {\kern 1pt} = {\kern 1pt} {\kern 1pt} \gamma {\kern 1pt} {\kern 1pt} \,\frac{P - \alpha }{\beta }$$

Above equation is a governing equation and can be solved by separating variables as under

3 $$\frac{dPSi}{{\frac{P - \alpha }{\beta }}}\,{\kern 1pt} {\kern 1pt} = {\kern 1pt} {\kern 1pt} {\kern 1pt} - {\kern 1pt} {\kern 1pt} \gamma {\kern 1pt} {\kern 1pt} d{\kern 1pt} {\kern 1pt} Si$$

Minus sign shows the decreasing physiological response against increased stress level. Equation 3 can be rewritten by introducing new constant a = 1/β, and b = α/β as below

4 $$\int {} \frac{dPSi}{{aP - b}}{\kern 1pt} {\kern 1pt} = {\kern 1pt} {\kern 1pt} {\kern 1pt} - {\kern 1pt} {\kern 1pt} {\kern 1pt} \gamma {\kern 1pt} {\kern 1pt} \int {{\kern 1pt} {\kern 1pt} d{\kern 1pt} Si}$$

General solution of Eq. 4 can be obtained as below

5 $$\frac{1}{\alpha }\ln {\kern 1pt} {\kern 1pt} {\kern 1pt} (aP - b) = - \gamma {\kern 1pt} {\kern 1pt} Si$$

6 $$\ln {\kern 1pt} {\kern 1pt} {\kern 1pt} {\kern 1pt} {\kern 1pt} (aP - b) = - {\kern 1pt} {\kern 1pt} {\kern 1pt} \alpha {\kern 1pt} {\kern 1pt} {\kern 1pt} \gamma {\kern 1pt} {\kern 1pt} Si$$

If λ = aγ, above Eq. 6 reduces to

7 $$\ln {\kern 1pt} {\kern 1pt} {\kern 1pt} {\kern 1pt} {\kern 1pt} (aPsi{\kern 1pt} {\kern 1pt} {\kern 1pt} - {\kern 1pt} {\kern 1pt} {\kern 1pt} b){\kern 1pt} {\kern 1pt} {\kern 1pt} {\kern 1pt} = {\kern 1pt} {\kern 1pt} {\kern 1pt} - {\kern 1pt} \lambda {\kern 1pt} {\kern 1pt} Si$$

*P*_Si_ is physiological response against silica dose of S

8 $$(aPsi{\kern 1pt} {\kern 1pt} {\kern 1pt} - {\kern 1pt} {\kern 1pt} {\kern 1pt} b){\kern 1pt} {\kern 1pt} {\kern 1pt} {\kern 1pt} = {\kern 1pt} {\kern 1pt} {\kern 1pt} e{\kern 1pt} {\kern 1pt}^{{ - \lambda {\kern 1pt} Si}}$$

9 $$P_{Si} = \frac{b}{a} + \frac{1}{a}e^{{{\kern 1pt} {\kern 1pt} - \lambda {\kern 1pt} {\kern 1pt} Si}}$$

10 $$Psi{\kern 1pt} {\kern 1pt} {\kern 1pt} {\kern 1pt} = {\kern 1pt} {\kern 1pt} \mu + \sigma {\kern 1pt} e{\kern 1pt}^{{ - {\kern 1pt} \lambda {\kern 1pt} {\kern 1pt} Si}}$$

11 $$Psi{\kern 1pt} {\kern 1pt} {\kern 1pt} {\kern 1pt} = {\kern 1pt} {\kern 1pt} {\kern 1pt} {\kern 1pt} \sigma {\kern 1pt} {\kern 1pt} (\rho + e{\kern 1pt}^{{{\kern 1pt} {\kern 1pt} - {\kern 1pt} {\kern 1pt} \lambda {\kern 1pt} {\kern 1pt} {\kern 1pt} Si}} )$$

where, ρ = μ/σ.

## Variations of photosynthetic response rate (P_t_), stomatal conductance and transpiration rate as a function of time (T) under silicon application for water stress mitigation

### **Hypothesis-II**

1. The rate of change of physiological responses (P) with respect to time (T), i.e. (dP/dT) is directly proportional to the expected maximum physiological response rate (Pm) under a given situation. Mathematically it can be written as below
  12 $$\frac{dP}{{dT}}\,{\kern 1pt} {\kern 1pt} \propto {\kern 1pt} {\kern 1pt} \,P_{m}$$
2. Rate of change of physiological response (P) with respect to time (T) i.e. (dP/dT) is also directly proportional to the expected difference between expected maximum physiological response rate and present physiological response rate under a given conditions and can be written as below
  13 $$\frac{dP}{{dT}}\,{\kern 1pt} {\kern 1pt} \propto {\kern 1pt} {\kern 1pt} \,(P_{m} - P)$$

Combining hypotheses 12 and 13, a general hypothesis can be written as below

14 $$\frac{dP}{{dT}}\,{\kern 1pt} {\kern 1pt} \propto {\kern 1pt} {\kern 1pt} \,P_{m} (P_{m} - P)$$

Introducing a proportionality constant ζ in Eq. 3 one will arrive at following governing equation

15 $$\frac{dP}{{dT}}\,{\kern 1pt} {\kern 1pt} = {\kern 1pt} {\kern 1pt} {\kern 1pt} {\kern 1pt} {\kern 1pt} \zeta {\kern 1pt} P_{m} \,(P_{m} - P)$$

Separating the variables and integrating,

16 $$\int {} \frac{dP}{{\left( {P_{m} {\kern 1pt} {\kern 1pt} {\kern 1pt} - {\kern 1pt} {\kern 1pt} {\kern 1pt} {\kern 1pt} {\kern 1pt} P} \right)}}\,{\kern 1pt} {\kern 1pt} = {\kern 1pt} {\kern 1pt} {\kern 1pt} \tau {\kern 1pt} {\kern 1pt} \int {dT}$$

where τ = ζ.P_m_, a new constant.

Integrating Eq. 16 one will obtain the following solution

17 $$- \ln {\kern 1pt} {\kern 1pt} {\kern 1pt} (P_{m} {\kern 1pt} {\kern 1pt} - {\kern 1pt} {\kern 1pt} {\kern 1pt} P){\kern 1pt} {\kern 1pt} {\kern 1pt} = {\kern 1pt} {\kern 1pt} {\kern 1pt} {\kern 1pt} {\kern 1pt} \tau {\kern 1pt} T + {\kern 1pt} {\kern 1pt} {\kern 1pt} {\kern 1pt} I$$

Which can be simplified as below

18 $$P = {\kern 1pt} {\kern 1pt} {\kern 1pt} P_{m} {\kern 1pt} {\kern 1pt} - {\kern 1pt} {\kern 1pt} {\kern 1pt} C{\kern 1pt} e^{{{\kern 1pt} - \tau {\kern 1pt} T}} {\kern 1pt}$$

where C = e^I^ and can be worked out by substituting initial conditions as T = 0, P = 0 as below

19 $$0 = {\kern 1pt} {\kern 1pt} {\kern 1pt} P_{m} {\kern 1pt} {\kern 1pt} - {\kern 1pt} {\kern 1pt} {\kern 1pt} C{\kern 1pt} e^{{{\kern 1pt} - \tau {\kern 1pt} {\kern 1pt} x{\kern 1pt} {\kern 1pt} 0}} {\kern 1pt}$$

20 $$C = {\kern 1pt} {\kern 1pt} {\kern 1pt} P_{m} {\kern 1pt} {\kern 1pt}$$

A generalized solution can be written as below

21 $$P_{t} = {\kern 1pt} {\kern 1pt} {\kern 1pt} P_{m} {\kern 1pt} {\kern 1pt} (1 - {\kern 1pt} {\kern 1pt} e^{{{\kern 1pt} - \tau {\kern 1pt} T}} )$$

### Statistical analyses

Statistical analyses were performed on physiological responses to silicon and water stress condition, depending on observations using CurveExpert 1.4 software.

## Results

**Fig. 1:**
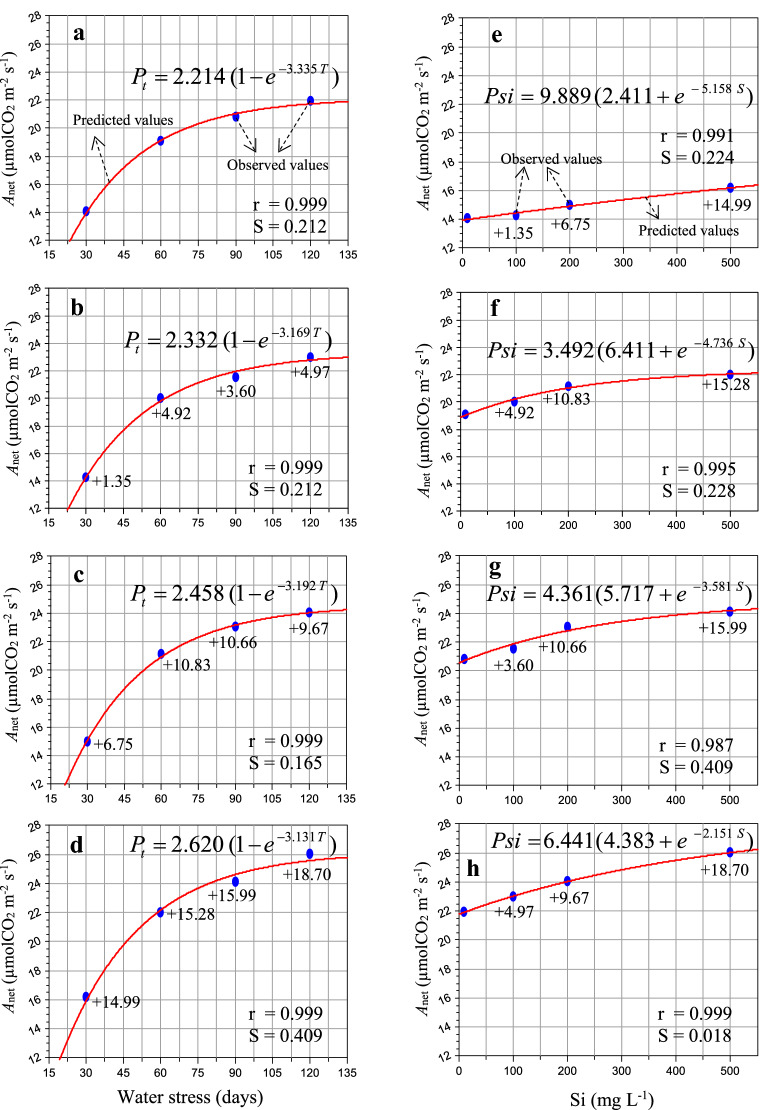
The change of net photosynthetic assimilation rate (*A*_net_—µmol CO_2_ m^−2^ s^−1^) to water stress condition (55–50% soil moisture, moderate, **a**–**d**) and different silicon levels (**e**–**h**) in sugarcane (*Saccharum officinarum* L. cv. GT 42) plants. Average mean values are each point (*n* = 5). Blue ovals denote the actual values and red lines show the predicted values. Parenthesis values indicate ( +) percent gain. S = standard error and r = correlation coefficient

Photosynthetic leaf gas exchange is the major process for all metabolic processes in plants. After soil irrigation of Si solution for 30, 60, 90 and 120 days, the net photosynthetic assimilation rate (*A*_net_), stomatal conductance to water vapor (gs) and rate of transpiration (E) were determined. It is clear from Fig. 1a–h, which showed significantly different between water stress (no Si) and Si (100, 200 and 500 mg L^−1^) at specific time intervals. The results showed that the *A*_net_ of sugarcane leaves was significantly enhanced by 1.35–18.70% by low to high Si concentrations, respectively, compared to the water-stressed plants (Fig. 1). The highest (%) gain was found in *A*_net_ of 500 mg L^−1^ Si (14.99–18.70%) as compare to 100 and 200 mg L^−1^ Si at 30, 60, 90 and 120 days after Si application (Fig. 1e–h). On the other hand, the *A*_net_ was found maximum (4.97 and 18.70%) at 120 days (Fig. 1b and d) except Fig. 1c.

**Fig. 2:**
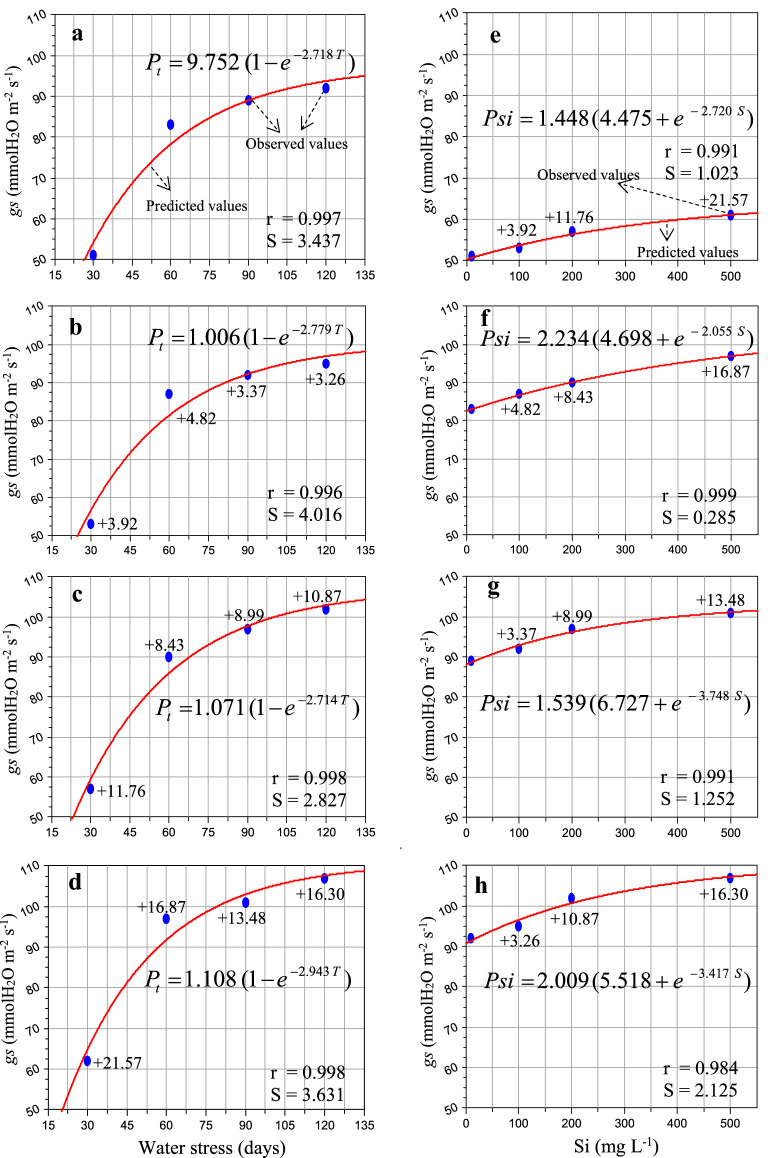
Response of stomatal conductance to water vapor (gs—mmol H_2_O m^−2^ s^−1^) to water stress condition (55–50% soil moisture, moderate,**a**–**d**) and different silicon levels (**e**–**h**) in sugarcane (*Saccharum officinarum* L. cv. GT 42) plants. Average mean values are each point (*n* = 5). Blue ovals denote the actual values and red lines show the predicted values. Parenthesis values indicate ( +) percent gain against stress. S = standard error and r = correlation coefficient

**Fig. 3:**
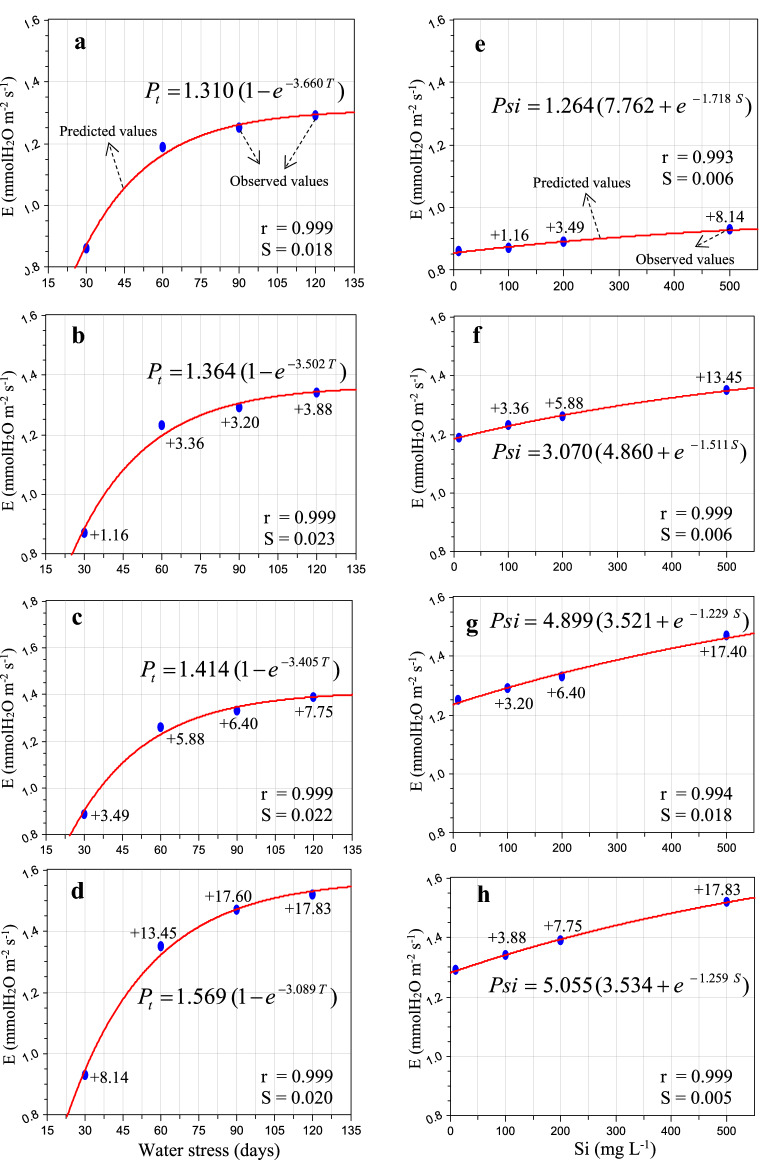
Effect of water stress **a**–**d**) and different silicon levels (**e**–**h**) on the rate of transpiration (mmol H_2_O m^−2^ s^−1^) in sugarcane (*Saccharum officinarum* L. cv. GT 42) plants. Average mean values are each point (*n* = 5). Blue ovals denote the actual values and red lines show the predicted values. Parenthesis values indicate ( +) percent gain against stress. S = standard error and r = correlation coefficient

Figure 2 showed that the low to high concentrations of Si (100 to 500 mg L^−1^) significantly enhanced gs of the sugarcane leaves by 3.26–21.57% (Fig. 2b–h) as compared to the stressed plants (no Si), respectively. Silicon enhanced the gs activity and improved and/or maintained biomass productivity. The highest (%) gain (13.48–21.57%) was found in 500 mg L^−1^ Si dose as compare to the lowest concentration (Fig. 2e–h ). On the other hand the maximum (%) gain was noticed in (11.76 and 21.57%) at 30 days (200 and 500 mg L^−1^) as compare to 60, 90 and 120 days after Si application (Fig. 2b–d). After soil irrigation of Si for specific time intervals, i.e. 30, 60, 90 and 120 days, the rate of transpiration was observed on the photosynthetically mature leaves of sugarcane (Fig. 3b–h). The results showed that the higher dose of Si (500 mg L^−1^) significantly enhanced the E response (8.14–17.83%) as compared to the stressed plants, respectively (Fig. 3). As shown in Fig. 3b–d, the E was found the highest (3.88, 7.75 and 17.83%) at 120 days after Si application as compared to 30, 60 and 90 days after water stress (Fig. 3b–d).

The data of *A*_net_, gs and E fit well within the derived equation, and their respective values of ‘r’ were observed to be in the range of 0.999–0.999, 0.996–0.998 and 0.999–0.999 and ‘S’ 0.165–0.409, 2.827–4.016 and 0.018–0.023 for water stress condition (Table 3). The *A*_net_, gs and E also fit well in the derived hypothesis equation with ‘r’ values ranging from 0.987 to 0.999, 0.984–0.999 and 0.993–0.999 and ‘S’ ranging from 0.018 to 0.409, 0.285–2.125, 0.005–0.018 for various levels of Si application (Table 4). The hypotheses were validated and developed models tested well with field observations.

**Table 3:** Model constants of net photosynthetic assimilation rate (*A*_net_), stomatal conductance to water vapour (gs) and rate of transpiration (E) subjected to water stress condition and Si application

Photosynthetic response	**S**tress (days)	Pm	τ	r	S
*A*_net_	30	2.214	3.335	0.999	0.212
	60	2.332	3.169	0.999	0.289
	90	2.458	3.192	0.999	0.165
	120	2.620	3.131	0.999	0.409
gs	30	9.752	2.718	0.997	3.437
	60	1.006	2.779	0.996	4.016
	90	1.071	2.714	0.998	2.827
	120	1.108	2.943	0.998	3.631
E	30	1.310	3.660	0.999	0.018
	60	1.364	3.502	0.999	0.023
	90	1.414	3.405	0.999	0.022
	120	1.569	3.089	0.999	0.020

*Pm* and *τ* Model constants, *r* Correlation coefficient, *S* Standard error, and 30, 60, 90 and 120 indicates days of stress

**Table 4:** Model constants of net photosynthetic assimilation rate (*A*_net_), stomatal conductance to water vapour (gs) and rate of transpiration (E) to Si application

Photosynthetic response	Silicon (mg L^−1^)	σ	ρ	ʎ	r	S
*A*_net_	0	9.889	2.411	5.158	0.991	0.224
	100	3.492	6.411	4.736	0.995	0.228
	200	4.361	5.717	3.581	0.987	0.409
	500	6.441	4.383	2.151	0.999	0.018
gs	0	1.448	4.475	2.720	0.991	1.023
	100	2.234	4.698	2.055	0.999	0.285
	200	1.539	6.727	3.748	0.991	1.252
	500	2.009	5.518	3.417	0.984	2.125
E	0	1.264	7.762	1.718	0.993	0.006
	100	3.070	4.860	1.511	0.999	0.006
	200	4.899	3.521	1.229	0.994	0.018
	500	5.055	3.534	1.259	0.999	0.005

*σ*, *ρ* and *ʎ* model constants, *r* Correlation coefficient, *S* Standard error, and 0, 100, 200 and 500 mg L^−1^ indicates Si concentration

## Discussion

Photosynthetic leaf gas exchange is the main source of all metabolic processes and biomass production in plants [2, 31]. Chlorophyll is an important pigment associated in photosynthetic capacity and plays a vital role in the absorption and transmission of light energy [10, 32]. Insufficient water causes a major loss in plant leaf area-expansion, photosynthetic pigments, which undeniably impairs and decrease photosynthetic CO_2_ assimilation rate, directly involved in plant growth and development [16, 18, 33]. Exogenous application of Si preserves photosynthetic pigments and a result increased and/ or improved photosynthetic performance in the plants cultivated during unfavourable environmental conditions [10, 14, 17, 18]. The present observations showed that the application of Si mitigates the harmful effects of water-deficit and enhanced the leaf canopy, leaf chlorophyll index in comparison to without Si under stress condition (Figs. 1, 2, 3). Similar findings are confirmed by the previous demonstrations of Farooq and Dietz [17], Shi et al. [14], Frew et al. [18], Verma et al. [2, 10].

For the water-stressed plants, stomatal closure is one of the first plant response to reduce loss of water, accompanied by a remarkable loss in gs and consequently, stomatal limitation of *A*_net_ [10, 11]. The impact of Si on leaf photosynthetic responses were highly dose-dependent (Figs. 1, 2, 3). Nevertheless, the application of Si also resulted in an enhancement on the rate of transpiration, possibly driven by the increased gs activity to maintain a steady state of photosynthetic CO_2_ assimilation rate subjected to water-stressed plants. Different relevant mechanisms linked with stress mitigation in higher plants have been noticed: enhanced untrastructural reinforcement [34], altered photosynthesis [12, 35, 36] and changes in gs activity [14, 37]. In the present study, the rate of transpiration in sugarcane leaves were enhanced by addition of Si subjected to limited water irrigation (Fig. 3b–h). Similar findings are in accordance with those measured in *Triticum aestivum, Sorghum bicolour, Oryza sativa* and *Solanum lycopersicum* [14, 37–39]. These findings suggest that the impact of Si on photosynthetic leaf gas exchange may be associated to plant families, varieties and duration of stress.

### Significance of bio-model hypothesis

The correlation coefficients (r) for each set of data such as *A*_net_, gs and E with and without Si application with water stress condition were found in the range of 0.984–0.999 (Tables 3 and 4). This means that the derived equation model explains the changes in photosynthetic capacity subjected to stress condition with various levels of Si and time perfectly well. Instrumental and human errors all together are within 2% limit. The developed models explain the rate of change of physiological responses with respect to Si application and time (stress duration) precisely. This may be directly proportional to the appropriate physiological responses against Si application for predicting the gains (%) of other plants in similar conditions, which can also be verified. The developed models may be useful to understand the nature of changes in major physiological response rates with respect to silicon applications and time.

## Conclusion

The results of the present study clearly demonstrated the capacity of different levels of Si to ameliorate the tolerance to water-deficit condition. The exogenous application of Si showed potential results by increasing photosynthetic leaf gas exchange activities and improving plant biomass. The present experiment demonstrates the appropriate concentration of Si can be applied to alleviate the harmful effects of water-stressed in sugarcane plants.

## Data Availability

Not available.

## Abbreviations

*A*_net_: Net photosynthetic assimilation rate
Gs: Stomatal conductance to water vapour
E: Rate of transpiration
PAR: Photosynthetic active radiation
Si: Silicon
